# Mechanisms of transcranial magnetic stimulation in the treatment of anorexia nervosa

**DOI:** 10.1192/bjo.2021.180

**Published:** 2021-06-18

**Authors:** Lydia Shackshaft

**Affiliations:** King's College London

## Abstract

**Aims:**

Severe and Enduring Anorexia Nervosa (SE-AN) is a challenging condition to treat, with limited therapeutic options, high morbidity, and the highest mortality rates of any psychiatric illness. Repetitive Transcranial Magnetic Stimulation (rTMS) is an emerging treatment option, as evidence demonstrates promising efficacy in improving mood and reducing core Anorexia Nervosa symptoms, as well as safety and tolerability to patients. We aimed to investigate the neurophysiological mechanisms of rTMS use in SE-AN patients by assessing changes in resting state functional connectivity, in the first functional neuroimaging analysis investigating rTMS effects in Anorexia Nervosa patients.

**Method:**

26 females with a current diagnosis of SE-AN received 20 sessions of sham or real high frequency rTMS (10 hertz) to the left dorsolateral prefrontal cortex in a randomised double-blind trial. Resting-state functional magnetic resonance imaging was performed before and after rTMS. Neural correlates of rTMS treatment were identified using a seed-based functional connectivity analysis with the left dorsolateral prefrontal cortex and bilateral amygdalae as regions of interest. Functional connectivity differences were analysed using t-contrasts in a mixed ANOVA (flexible factorial analysis) to assess interactions between treatment group (real rTMS vs sham) and time-point (pre or post TMS).

**Result:**

No statistically significant changes in resting-state functional connectivity were observed post-rTMS compared to baseline in participants receiving active rTMS compared to sham. Increased functional connectivity between the left amygdala and left pre-supplementary motor area was observed to reach cluster-wise significance (PFWE < 0.05). However, after Bonferroni correction for multiple comparisons (3 seed regions), this did not reach the significance threshold PFWE <0.017.

**Conclusion:**

This study highlights the need for further investigation of neurophysiological mechanisms, including resting-state functional connectivity modulation, resulting from rTMS to the dorsolateral prefrontal cortex in SE-AN patients. This requires higher powered studies to account for heterogeneity in treatment response. We have provided some indication that high frequency rTMS may have therapeutic benefit in SE-AN by modification of functional connectivity between prefrontal and limbic brain regions, resulting in improved top-down cognitive control over emotional processing and ability to enact goal-directed behaviours, enabling secondary reductions in eating disorder behaviours.

